# Neuroprotection by *Anethum graveolens* (Dill) Seeds and Its Phytocompounds in SH-SY5Y Neuroblastoma Cell Lines and Acellular Assays

**DOI:** 10.3390/ijms25137104

**Published:** 2024-06-28

**Authors:** Himadri Sharma, Hyewon Yang, Niti Sharma, Seong Soo A. An

**Affiliations:** Department of Bionano Technology, Gachon Bionano Research Institute, Gachon University, 1342 Seongnam-daero, Sujung-gu, Seongnam-si 461-701, Gyeonggi-do, Republic of Korea

**Keywords:** dill, apiole, carvone, neuroprotection, H_2_O_2_-induced oxidative stress in SH-SY5Y, anti-acetylcholine esterase activity, anti-Aβ fibrilization/oligomerization

## Abstract

Neurodegeneration diseases (NDs) are a group of complex diseases primarily characterized by progressive loss of neurons affecting mental function and movement. Oxidative stress is one of the factors contributing to the pathogenesis of NDs, including Alzheimer’s disease (AD). These reactive species disturb mitochondrial function and accelerate other undesirable conditions including tau phosphorylation, inflammation, and cell death. Therefore, preventing oxidative stress is one of the imperative methods in the treatment of NDs. To accomplish this, we prepared hexane and ethyl acetate extracts of *Anethum graveolens* (dill) and identified the major phyto-components (apiol, carvone, and dihydrocarvone) by GC-MS. The extracts and major bioactives were assessed for neuroprotective potential and mechanism in hydrogen peroxide-induced oxidative stress in the SH-SY5Y neuroblastoma cell model and other biochemical assays. The dill (extracts and bioactives) provided statistically significant neuroprotection from 0.1 to 30 µg/mL by mitigating ROS levels, restoring mitochondrial membrane potential, reducing lipid peroxidation, and reviving the glutathione ratio. They moderately inhibited acetylcholine esterase (IC_50_ dill extracts 400–500 µg/mL; carvone 275.7 µg/mL; apiole 388.3 µg/mL), displayed mild anti-Aβ_1–42_ fibrilization (DHC 26.6%) and good anti-oligomerization activity (>40% by dill-EA, carvone, and apiole). Such multifactorial neuroprotective displayed by dill and bioactives would help develop a safe, low-cost, and small-molecule drug for NDs.

## 1. Introduction

Neurodegenerative diseases (NDs) are a group of diseases that result from advancing deterioration in the structure and function of neurons. The worldwide statistical data speculated that over 152 million people will be affected by ND-related issues by 2050 [1]. NDs include Alzheimer’s disease (AD), Parkinson’s disease (PD), Huntington’s disease (HD), amyotrophic lateral sclerosis (ALS), prion diseases, etc. In NDs, different regions in the brain are affected but some characteristics like proteinopathy and induced cell death are common [2,3]. Hence, therapeutic interventions against one ND can improve symptoms of other ND as well. Additionally, oxidative stress and neuroinflammation are identified as major contributors to the disease [4,5]. The cause of oxidative stress is an abundance of reactive oxygen species (ROS) due to overproduction or decreased elimination in the system. Increased ROS reacts and damages cellular components hastening inflammation, tau phosphorylation, and apoptosis in neurons [6]. Thus, protecting the cells from oxidative stress would be a significant approach to treating NDs. To date, no remedy effectively cures complex NDs, and available symptomatic medications entail significant side effects. Therefore, a plant-based potential multi-targeted therapy would be valuable in treating NDs. Plants are a rich source of antioxidants and other bioactive components that can target complex pathological pathways of neurodegeneration in various ways.

*Anethum graveolens* L. (dill; Family Apiaceae) is an aromatic herb cultivated worldwide. The dill seeds have an intense fragrance and are used as a spice for flavoring the food. On crushing, the leaves and fruits release a strong fragrance due to aromatic compounds in the essential oils rich in α-phellandrene, limonene, dill apiole, carvacrol, carvone, and *p*-cymene [7,8]. Both the leaves and fruits have therapeutic properties like antioxidant, anti-cancer [9], anti-inflammatory [10], antinociceptive [11], anti-microbial [12], anti-parasitic [13], carminative and diuretic properties [14]. It is also effective in treating irritable bowel syndrome [15], hyperlipidemia [16], diabetes [17], etc. Furthermore, dill essence [18] and combined extract [19,20] reportedly improved cognition in animal models. In the central nervous system (CNS), cholinergic transmission (acetylcholine; ACh) has a vital role in maintaining neuronal plasticity, and cell survival, thus compromised cholinergic signaling results in memory deficits [21].

To gain insight into the neuroprotective mechanisms of dill, we evaluated the effects of dill extract (hexane and ethyl acetate) and its bioactive components on hydrogen peroxide-induced oxidative stress in human neuroblastoma SH-SY5Y cells. These cells are widely used in neuroscience as in vitro models, particularly for studying neuroprotective mechanisms. The hydrogen peroxide (H_2_O_2_) treatment was employed to induce oxidative stress in the SH-SY5Y cells, mimicking conditions observed in NDs. Additionally, acetylcholine esterase enzyme (AChE) inhibition, anti-oligomerization, and anti-fibrillation activity were also studied to identify various other mechanisms for neuroprotection.

## 2. Results

### 2.1. Phytochemical Estimation and Antioxidant Potential of Dill Extract

Total phenolic (TPC) and flavonoids (TFC) were estimated in the extracts using colorimetric assays. The content of phenolics was calculated from the regression equation of the calibration curve (R^2^ = 0.995, *y* = 0.046*x* + 0.0059), expressed in gallic acid equivalent (GAE) as milligrams per gram of the extract or fraction (mg GAE/g extract). TPC of the extracts was estimated to be 16.20 ± 2.88 mg GAE/g and 43.55 ± 3.42 mg GAE/g in dill-H (hexane) and dill-EA (ethyl acetate), respectively. The flavonoid content was calculated from the regression equation of the calibration curve (R^2^ = 0.994, *y* = 0.0432*x* + 0.0051), expressed in quercetin equivalent (QE) as milligrams per gram of the extract or fraction (mg QE/g extract). TFC in the dill-EA (18.94 ± 3.45 mg QE/g) was higher than the dill-H (8.69 ± 2.05 mg QE/g). The antioxidant potential of the extracts was evaluated using DPPH, ABTS, and FRAP assays. In the DPPH assay, the percent radical scavenging activity observed was 37.11 ± 0.23% and 53 ± 0.71% for dill-H and dill-EA, respectively. A similar activity like dill-H was observed for the pure compounds (apiole: 29.73 ± 2.27%; carvone: 37.05 ± 0.25%; DHC: 36.85 ± 1.51%) in the assay. Ascorbic (10 μg/mL) displayed 85.43 ± 1.02% activity as a positive control in the assay. In the ABTS assay, dill-EA (45.72 ± 0.64%) displayed better radical scavenging activity than dill-H (37.17 ± 0.14%). The pure compounds (apiole: 20.71 ± 0.26%; carvone: 20.02 ± 0.18%; DHC: 17.53 ± 0.47%) exhibited lower activity as compared to the extracts. Positive control, quercetin (10 μg/mL) displayed 97.55 ± 0.05% activity in the assay. In the FRAP assay reduction of Fe^3+^ to Fe^2+^ in the presence of antioxidants is measured. Here also dill-EA exhibited a better FRAP value (103 ± 1.03 µM Fe^2+^/g) compared to dill-H (68.48 ± 1.2 µM Fe^2+^/g). The FRAP activity of pure compounds was lower than that of the extracts (ranging from 42.41 to 45.22 µM Fe^2+^/g). Ascorbic acid positive control (10 μg/mL) displayed 142.83 ± 0.4 1% activity in the assay. The results were summarized in Supplementary Table S1.

### 2.2. GC–MS Analysis

**Figure 1:**
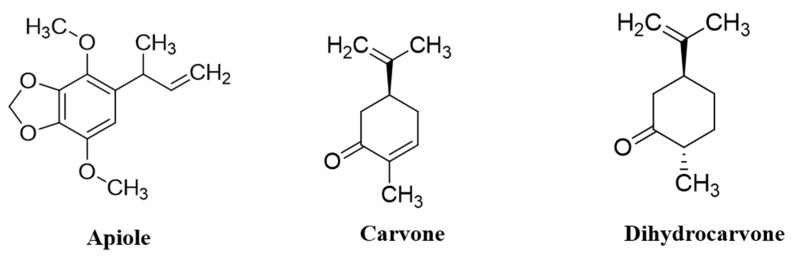
The main phytocompounds identified in the dill extract.

The GC–MS chromatogram of hexane and ethyl acetate extracts of dill identified three peaks each. The peaks were identified by comparing retention time, peak area (%), height (%), and mass spectral fragmentation to that of the known compounds in the NIST library (Supplementary Figure S1). The main components identified were 19.9% cyclohexanone,2-methyl-5-(1-methylethenyl)- [syn. DHC; dihydrocarvone], 26.55% D-carvone, and 52.46% apiole in dill-H. Meanwhile, 9.26% cyclohexanone,2-methyl-5-(1-methylethenyl)-, 14.9% D-carvone, and 53.61% apiole were identified in dill-EA (Figure 1).

### 2.3. In Vitro Acetylcholinesterase Inhibitory Activity

**Figure 2:**
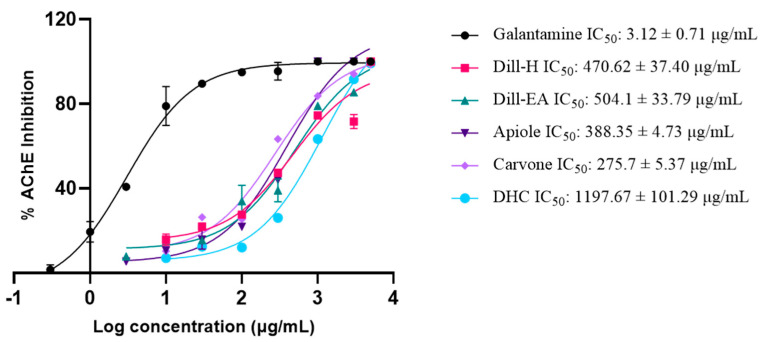
IC_50_ curves of dill extracts, apiole, carvone, and DHC with inhibitor control (galantamine) against AChE (*Electrophorus)*. The IC_50_ values were calculated using GraphPad Prism 10. The values were expressed as a mean of three experiments ± SEM.

Acetylcholinesterase (AChE; E.C.3.1.1.7) is a cholinergic enzyme generally present at the postsynaptic neuromuscular junctions and hydrolyses acetylcholine (ACh), an important neurotransmitter. In AD patients, the level of ACh declines in the synaptic junction hence, inhibition of AChE is desirable to maintain the normal ACh levels. Therefore, the extracts and the phytocompounds were screened for anti-AChE activity and the IC_50_ values (half maximal inhibitory concentration) were calculated using galantamine hydrobromide as inhibitor control. The IC_50_ value of dill-H (470.62 ± 37.40 μg/mL) was lower than the dill-EA (504.10 ± 33.79 μg/mL) extract. Among the phytocompounds, D-carvone displayed better inhibitory activity (IC_50_: 275.70 ± 5.37 μg/mL or 1.83 mM) compared to apiole (IC_50_: 388.35 ± 4.73 μg/mL or 1.74 mM), and DHC (IC_50_ > 1197.67 ± 101.29 μg/mL or 7.86 mM) (Figure 2). The IC_50_ value of galantamine was 3.12 ± 0.71 μg/mL (8.47 μM), similar to the previously reported value of 4.31 μg/mL.

The K_m_ and V_max_ values (Table 1) were calculated using a non-linear fit (Michaelis–Menten equation) and the inhibition curves (with and without extract/phytocompound) were plotted using the linear regression (Lineweaver–Burk plot) on GraphPad Prism 10. We have not conducted kinetic analysis on DHC as it was a weak AChE inhibitor (IC_50_ > 1 mg/mL). The competitive inhibition pattern observed by the extracts and the phytocompounds for the enzyme (Supplementary Figure S2) suggested that they compete with the substrate for binding to the enzyme’s active site. The Ki value is the dissociation constant describing the binding affinity between the inhibitor and the enzyme. K_i_ (inhibitor constant) value for the competitive inhibition was calculated from the formula IC_50_ = K_i_ (1 + [S]/K_m_) [22]. From the Ki values (Table 1), carvone had the lowest Ki value (270 μg/mL or 1.79 mM) compared to others suggesting that carvone has the strongest binding affinity for the enzyme, followed by apiol (382 μg/mL or 1.71 mM) and dill-H (464 μg/mL).

### 2.4. Aβ -Fibrilization and Oligomerization Inhibition by Dill

**Figure 3:**
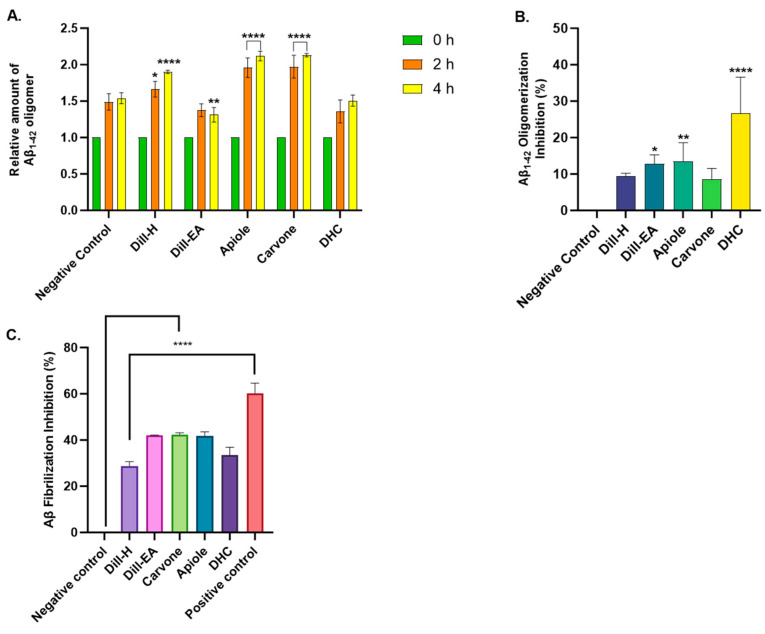
Aβ oligomerization and fibrilization inhibition by dill extracts, apiole, carvone, and DHC. (**A**) The relative amount of Aβ oligomers at 0, 2, and 4 h. (**B**) Aβ oligomerization inhibition. (**C**) ThT anti-fibrilization assay. Phenol red (50 μM) was used as a positive control. All data are expressed as mean ± SEM (*n* = 3). A significant difference * (*p* < 0.05), ** (*p* < 0.01), and **** (*p* < 0.0001) using the two-way ANOVA (**A**) and one-way ANOVA (**B**,**C**) followed by Dunnett’s post hoc was observed in the percent oligomerization reduction vs. the negative control (Buffer + Aβ).

The multimeric detection system (MDS) was used to investigate the effect of dill on Aβ oligomerization. The results were calculated based on the zero h value at which all samples and control signals were set at 1.0. The oligomerization reduction at 2 h was statistically significant in dill-H (* *p* < 0.05), and apiole and carvone (**** *p* < 0.0001). A non-significant oligomerization reduction was observed in dill-EA and DHC. After 4 h of incubation, a statistically significant oligomerization reduction (*** *p* < 0.0001) was observed in the case of dill-H, apiole, and carvone except for dill-EA (* *p* < 0.01) (Figure 3A). The compounds may inhibit Aβ oligomerization at different times due to structural differences affecting their interaction with the protein. Additionally, the compounds may degrade with time [23].

Figure 3B displays the percent oligomerization inhibition by dill. The statistical significance was evaluated compared to the negative control. The extracts showed weak inhibition in the assay (dill-H: 7.53 ± 3.27%, non-significant, and dill-EA: 12.82 ± 1.74%; * *p* < 0.05). Among the bioactives; DHC (26.62 ± 8.62%; **** *p* < 0.0001) showed more potent oligomerization inhibition as compared to apiole (13.53 ± 4.4%; ** *p* < 0.01) and carvone (8.12 ± 2.68%, non-significant).

Inhibition of Aβ fibrilization by the extracts and the phytocompounds was analyzed by ThT assay. The binding of ThT to the β-sheet of amyloid fibrils increases the fluorescence thus it is used to monitor fibril formation [6]. The samples (500 μg/mL) were screened for anti-fibrilization potential using phenol red as the positive control. The results were statistically significant (^####^ *p* < 0.0001) compared to the negative control (Buffer + Aβ). Dill-H exhibited 28.77 ± 1.83% inhibitory activity while dill-EA (42.05 ± 0.14%), carvone (42.37 ± 0.76%), apiole (41.85 ± 1.73%), and DHC (33.44 ± 3.43%) showed potent inhibitory activity. The inhibition exhibited by phenol red (60.3 ± 4.29%) was similar to a previously reported value [24] Figure 3C.

### 2.5. Cytotoxic Effect of Dill Extracts and Its Bioactive Compounds

The cellular viability was estimated in the SH-SY5Y neuroblastoma cell line after 24 h of treatment with different concentrations of the extracts/ phytocompounds (1, 10, 30 µg/mL) using WST-8 dye. As no cytotoxicity was observed up to 30 µg/mL in both cases (Supplementary Figure S3) the subsequent cell culture experiments were conducted using this as maximum concentration.

### 2.6. Protective Effect of Dill Extracts and Its Bioactive Compounds against H_2_O_2_-Induced Oxidative Stress

**Figure 4:**
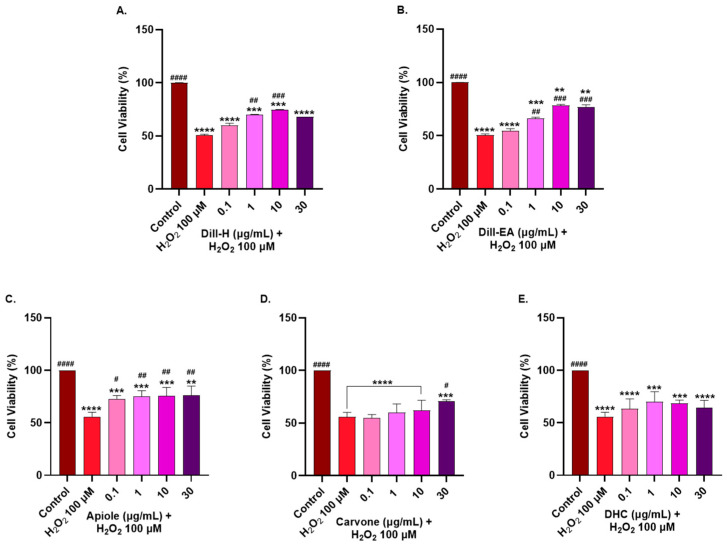
Neuroprotective effects of (**A**) dill-H, (**B**) dill-EA, (**C**) apiole, (**D**) carvone, and (**E**) DHC in H_2_O_2_-induced neuroblastoma SH-SY5Y cells at 0.1, 1, 10, and 30 µg/mL. The SH-SY5Y cells were preincubated with the extracts for 12 h, followed by 1 h of H_2_O_2_ (100 µM) treatment. The results indicate % cell viability vs. the control cells mean ± SEM (*n* = 3). A significant difference ^#^ (*p* < 0.05), **^/##^ (*p* < 0.01), ***^/###^ (*p* < 0.001) and ****^/####^ (*p* < 0.0001), using one-way ANOVA followed by Dunnett’s test, was observed in the % of cell viability vs. untreated cells (*) and H_2_O_2_ treated cells (^#^).

The neuroprotective effects of the extracts/phytocompounds were assessed by H_2_O_2_-induced oxidative stress in the SH-SY5Y cells. Around 50% cell survival after 1 h treatment with H_2_O_2_ (100 µM) was observed in our preliminary experiment. Therefore, this H_2_O_2_ concentration was used to induce stress in SH-SY5Y cells pre-treated with the extracts for 12 h. Dill-H displayed a significant increase in cell viability (70.26 ± 0.01%) at 1 µg/mL (^#^ *p* < 0.05) and 10 µg/mL (74.74 ± 0.04%; ^##^ *p* < 0.01), as compared to the H_2_O_2_ control. At 30 µg/mL, the cell viability decreased (68.09 ± 0.00%) compared to 10 µg/mL. Dill-EA provided better neuroprotection than dill-H at 1 µg/mL (66.44 ± 1.04%; ^#^ *p* < 0.01) and 10–30 µg/mL (78.43 ± 1.00% and 77.01 ± 2.06%; ^###^ *p* < 0.001) (Figure 4). Apiole exerted better neuroprotective activity than the other two pure compounds and increased significantly (72.82 ± 3.13%; ^#^ *p* < 0.05) at 0.1 µg/mL. The increase in cell viability was statistically significant (^##^ *p* < 0.01) and almost constant (~75%) at higher concentrations (1–30 µg/mL), as compared to the H_2_O_2_ control (Supplementary Figure S4). Carvone displayed significant (70.50 ± 1.67%; ^#^ *p* < 0.05) neuroprotection at 30 µg/mL only while DHC non-significantly increased the cell viability at all concentrations (0.1–30 µg/mL).

### 2.7. Dill and Its Phytocompounds Mitigated H_2_O_2_-Induced ROS Generation

**Figure 5:**
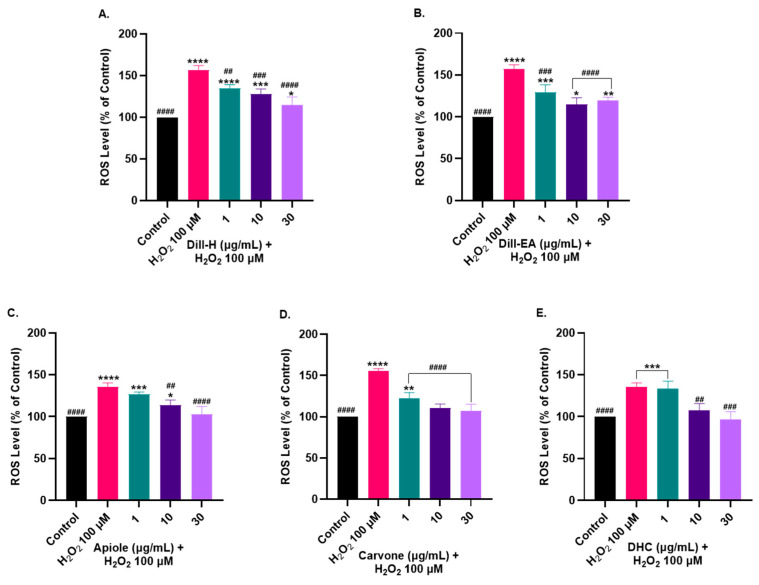
Effect of dill extracts, and phytocompounds on H_2_O_2_-induced ROS production in SH-SY5Y cells. The SH-SY5Y cells were preincubated with the (**A**) dill-H, (**B**) dill-EA, (**C**) apiole, (**D**) carvone, and (**E**) DHC at different concentrations (1, 10, and 30 µg/mL) for 12 h, followed by 2 h of H_2_O_2_ (100 µM) treatment. The results indicate the ROS level (%) in the control (untreated cells) and treated cells. Values are mean ± SEM (*n* = 3). The data were analyzed by one-way ANOVA followed by Dunnett’s test. A significant difference * (*p* < 0.05), **^/##^ (*p* < 0.01), ***^/###^ (*p* < 0.001), and ****^/####^ (*p* < 0.0001) was observed in the % ROS vs. untreated cells (*) and H_2_O_2_ treated cells (^#^).

To access the intracellular reactive oxygen species (ROS) scavenging activity of the extracts, the SH-SY5Y cells were pre-treated with the extracts for 12 h, followed by 2 h exposure to H_2_O_2_ (100 µM). The ROS production in the cells was monitored by a fluorescent dye (H2DCFDA), which is oxidized to DCF by ROS. The SH-SY5Y cells treated with H_2_O_2_ generated 156.96 ± 5.07% ROS compared to the untreated cells (100%). Pre-treatment of the cells with extracts resulted in a significant dose-dependent decrease in ROS production in dill-H (134.84 ± 4.15% at 1 µg/mL, ^##^ *p* < 0.01; 127.86 ± 6.04% at 10 µg/mL, ^###^ *p* < 0.001; and 115.13 ± 9.26% at 30 µg/mL, ^####^ *p* < 0.0001) (Figure 5A) and dill-EA (129.20 ± 9.02% at 1 µg/mL, ^#^ *p* < 0.05; 114.95 ± 7.84% at 10 µg/mL, ^##^ *p* < 0.01; and 110.9 ± 3.3% at 30 µg/mL, ^##^ *p* < 0.01) (Figure 5B).

Among the pure compounds, apiole and DHC displayed similar results with significant dose-dependent reduction in ROS [10 µg/mL (113.63 ± 6.25%; ^##^ *p* < 0.01), and 30 µg/mL (102.92 ± 9.11%; ^####^ *p* < 0.0001) for apiole and 10 µg/mL (107.37 ± 8.26%; ^##^ *p* < 0.01), and 30 µg/mL (96.73 ± 9.11%; ^###^ *p* < 0.001) for DHC] (Figure 5C,E). Carvone performed better as it significantly (^####^ *p* < 0.0001) reduced ROS production at 1 µg/mL (122.82 ± 6.63%), 10 µg/mL (110.70 ± 4.90%), and 30 µg/mL (107.11 ± 8.34%) (Figure 5D).

### 2.8. Dill Extract and Phytocompounds Improved Mitochondrial Membrane Potential

Oxidative stress and mitochondrial dysfunction have been implicated in the pathogenesis of several neurodegenerative diseases. The overproduction of reactive oxygen species (oxidative stress) can damage the mitochondrial respiratory chain, alter membrane potential (MMP; ΔΨm), and influence Ca^2+^ homeostasis.

**Figure 6:**
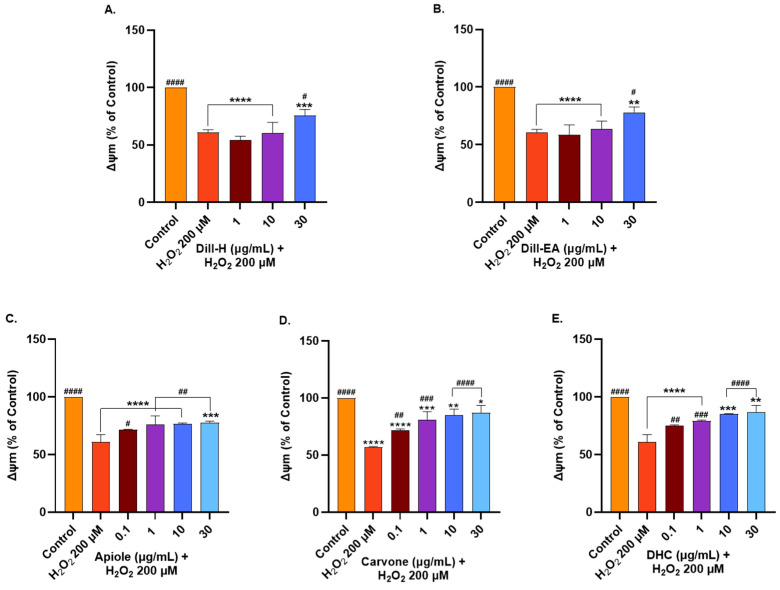
Mitochondrial membrane potential in SH-SY5Y cells pre-treated with (**A**) dill-H, (**B**) dill-EA, (**C**) apiole, (**D**) carvone, and (**E**) DHC followed by 200 μM H_2_O_2_ treatment for 2 h. The results indicate % ∆Ψm vs. the control cells (untreated cells). Values are mean ± SEM (*n* = 3). The data were analyzed by one-way ANOVA followed by Dunnett’s test. A significant difference *^/#^ (*p* < 0.05), **^/##^ (*p* < 0.01), ***^/###^ (*p* < 0.001), and ****^/####^ (*p* < 0.0001) was observed in the % cell viability vs. untreated cells (*) and H_2_O_2_ treated cells (^#^).

We used H_2_O_2_ to induce oxidative stress in SH-SY5Y cells and the ΔΨm was monitored using tetramethylrhodamine, an ethyl ester (TMRE) fluorescent dye that has an affinity for active mitochondria. The ΔΨm decreased in the depolarized membrane due to the inability to sequester the dye properly. In our study, a 50% reduction in the ΔΨm was observed in the untreated cells at 200 μM H_2_O_2_ hence this concentration was used for further examination. The cells were pre-treated with the extract/bioactives for 12 h followed by 200 μM H_2_O_2_ treatment for 2 h. The extracts behaved similarly by increasing MMP significantly (^#^ *p* < 0.05) at the highest dose (~75–78% at 30 µg/mL) whereas the lower concentrations (1 and 10 µg/mL) had no significant effect on MMP (Figure 6A,B).

The pure compounds displayed a significant dose-dependent increase in MMP from 0.1 µg/mL and the results for carvone and DHC were statistically more significant than apiole (Figure 6C–E). Apiole significantly (^#^ *p* < 0.05) increased the MMP at 0.1 µg/mL (71.53 ± 0.37%) after which it changed slightly from 75 to 77% (^##^ *p* < 0.001) at higher concentrations (1–30 µg/mL) (Figure 6C). Carvone pre-treatment significantly dose-dependently increased the MMP at 0.1 µg/mL (71.61 ± 0.70%; ^##^ *p* < 0.01), 1 µg/mL (80.80 ± 1.20%; ^###^ *p* < 0.0001), 10 µg/mL (85.18 ± 4.96%; ^####^ *p* < 0.0001), and 30 µg/mL (87.19 ± 6.26%; ^####^ *p* < 0.0001) (Figure 6D). DHC had a similar effect as carvone in increasing MMP with 74.94 ± 1.20% (^##^ *p* < 0.01) at 0.1 µg/mL, 79.26 ± 0.56% (^###^ *p* < 0.001) at 1 µg/mL, 85.21 ± 0.19% (^####^ *p* < 0.0001) at 10 µg/mL, and 86.91 ± 5.47% (^####^ *p* < 0.0001) at 30 µg/mL (Figure 6E).

### 2.9. Dill Extract and Bioactive Compounds Restored Oxidative Stress Markers Altered by H_2_O_2_-Induced Oxidative Stress

Subsequently, the effect of pre-treatment with dill extracts and the major bioactives (apiole, carvone, and DHC) was studied on the H_2_O_2_-induced oxidative stress-affected parameters (lipid peroxidation, glutathione) in the SH-SY5Y cells.

#### 2.9.1. Restoration of Glutathione Levels

The pre-treatment of SH-SY5Y cells with varying concentrations of dill extract (1, 10, and 30 μg/mL) and bioactives (0.1, 1, and 10 μg/mL) for 24 h before 6 h incubation with H_2_O_2_ (100 μM) resulted in a significant dose-dependent increase in GSH level (Supplementary Figure S5). Dill-EA performed significantly better at 1 µg/mL (^####^ *p* < 0.0001) compared to dill-H at the same concentration. At 10 and 30 µg/mL both the extracts behaved similarly (^####^ *p* < 0.0001) in restoring GSH level. All three bioactives showed similar dose-dependent responses at all concentrations (^####^ *p* < 0.0001). The oxidized glutathione (GSSG) levels were also measured (Supplementary Figure S6). The H_2_O_2_-induced stress raised intracellular GSSG levels. In our study, dill extracts significantly (^####^ *p* < 0.0001) reduced GSSG levels at 10 and 30 μg/mL. In the case of apiole and DHC, a significant decrease in GSSG was observed at 1 μg/mL and 10 μg/mL. However, DHC showed substantial reduction only at 10 μg/mL (^#^ *p* < 0.05).

**Figure 7:**
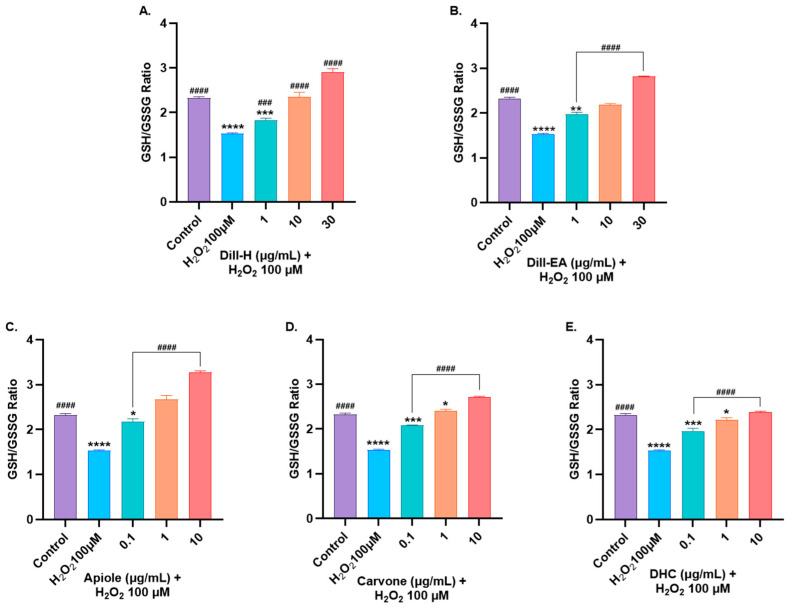
GSH/GSSG ratio in SH-SY5Y cell lysate exposed to 100 μM H_2_O_2_ for 6 h after 24 h of pre-treatment with (**A**) dill-H, (**B**) dill-EA, (**C**) apiole, (**D**) carvone, and (**E**) DHC. The results indicate the GSH/GSSG ratio in treated and control cells (untreated cells). Values are mean ± SEM (*n* = 3). The data were analyzed by one-way ANOVA followed by Dunnett’s test. A significant difference of * (*p* < 0.05), ** (*p* < 0.01), ***^/###^ (*p* < 0.001), and ****^/####^ (*p* < 0.0001), was observed in comparison to untreated cells (*) and H_2_O_2_ treated cells (^#^).

Reduced glutathione (GSH) is one of the most significant ROS scavengers, and its ratio with oxidized glutathione (GSSG) is a crucial indicator of cell health. We observed a significant dose-dependent increase in the GSH: GSSG ratio towards the normal state at all the tested concentrations of extracts and pure compounds ^(####^ *p* < 0.0001) except for dill-H at 1 μg/mL (^###^ *p* < 0.001) (Figure 7). Slightly better significant activity displayed by dill-EA than dill-H might be due to the higher ratio of bioactives in it.

#### 2.9.2. Attenuation of MDA Level by Dill Extract

MDA is the most common biomarker for lipid peroxidation and is directly proportional to the oxidative injury. Lipid peroxidation refers to the degradation of lipids resulting from oxidative damage.

**Figure 8:**
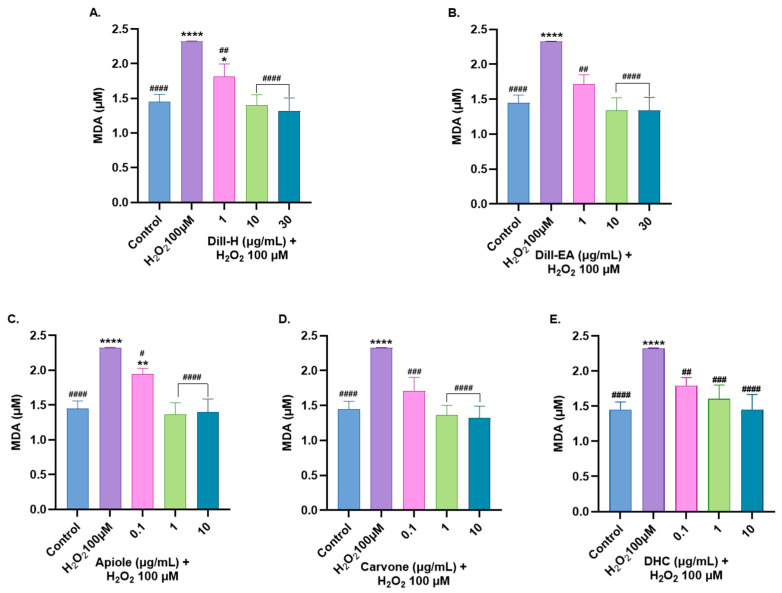
Malondialdehyde (MDA) content in SH-SY5Y cells lysate exposed to 100 μM H_2_O_2_ for 6 h after 24 h pre-treatment with (**A**) dill-H, (**B**) dill-EA, (**C**) apiole, (**D**) carvone, and (**E**) DHC. The results indicate MDA (μM) in treated and control cells (untreated cells). Values are mean ± SEM (*n* = 3). The data were analyzed by one-way ANOVA followed by Dunnett’s test. A significant difference *^/#^ (*p* < 0.05, **^/##^ (*p* < 0.01), ^###^ (*p* < 0.001), and ****^/####^ (*p* < 0.0001) was observed in comparison to untreated cells (*) and H_2_O_2_ treated (^#^) cells.

In the present study, dill extracts decreased MDA content in a statistically significant dose-dependent manner with complete attenuation at 10 and 30 µg/mL (~1.35 µM; ^####^ *p* < 0.0001) compared to the control (Figure 8). Carvone displayed more significant results compared to apiole and DHC. It decreased MDA levels significantly (1.71 µM; ^###^ *p* < 0.001) at 0.1 µg/mL followed by a complete reduction in MDA levels at 1 and 10 µg/mL (~1.33 µM; ^####^ *p* < 0.0001). DHC decreased MDA levels in a significant dose-dependent manner; 0.1 µg/mL (1.79 µM; ^##^ *p* < 0.01), 1 µg/mL (1.60 µM; ^###^ *p* < 0.001), and 10 µg/mL (1.45 µM; ^####^ *p* < 0.0001). Apiole reduced MDA level significantly at 0.1 µg/mL (1.94 µM; ^#^ *p* < 0.05) and the complete reduction was observed at 1 and 10 µg/mL (~1.38 µM; ^####^ *p* < 0.0001).

## 3. Discussion

The present study described the neuroprotective potential of dill extract and its key phytocompounds in the oxidative stress model using human neuroblastoma cell lines (SH-SY5Y). In the phytochemical analysis and antioxidant activity measurements, dill-EA performed better than dill-H due to higher TPC and TFC. The phenols and flavonoids are known to scavenge free radicals [25] and protect against oxidative stress. A positive correlation between phenolic content and antioxidant potential has been reported [23]. A similar antioxidant potential was observed for the dill tablet [26]. This property is commonly associated with reductones that provide hydrogen atoms, leading to the breaking of the free radical chain [27]. Previously, TPC and TFC in the methanolic extract were reported as 69.87 mg GAE/100 g dw and 49.10 mg CE/100 g dw, respectively, with 81.52% DPPH activity [28]. The difference in the obtained values can be related to variable varieties and extraction solvents.

GC-MS identified apiole, carvone, and DHC as the major phytocompounds. Dill herb oil has been approved as GRAS by the US FDA for its use in the food industry as a flavoring and seasoning agent [29]. DHC is a colorless, oily monoterpenoid with *p*-menthane consisting of the cyclohexane ring with a methyl group at position 1 and a 2-methyl-propyl group at position 4. D-carvone is a monoterpene ketone present as a volatile component of essential oils and exhibits numerous pharmacologic properties like antimicrobial, antidiabetic, antioxidant, anti-inflammatory, anticancer, and neurological activities [30]. Its LD_50_ has been reported as 1640 mg/kg in rodent models [31]. Carvone can cross BBB and exhibit drug-like properties according to Lipinski’s rule of five [32]. On the other hand, apiole is a benzodioxole and has acaricidal [33], anti-inflammatory [34], gastroprotective [35], and anticancer [36] activities. However, it lacks significant BBB permeability [32]. Chemically, apiole is a benzodioxole while carvone and DHC are monoterpenoids with potent antioxidant activity [37,38]. The presence of an unsaturated hydroxyl group and a conjugated double bond in carvone provides greater free radical scavenging activity [38,39]. Apiol has two electron-donating methoxy groups which increases the stability of the benzene ring and hence increases radical scavenging activity [40]. The synergistic action of these phytocompounds provided antioxidant properties to the dill extracts. The higher apiole content in dill-EA might contribute to its improved antioxidant activity.

The anti-AChE potential of the dill extracts and the key phytocompounds were investigated in vitro using AChE from *E. electricus*. The plant extracts with an anti-AChE activity have been classified as potent (>50% inhibition), moderate (30–50% inhibition), and low activity (<30% inhibition) at 100 μg/mL [41]. In the preliminary screening, dill extracts and bioactives at 100 μg/mL moderately inhibited AChE. From the experimental results, dill-EA (504.1 ± 33.79 μg/mL) had a slightly higher IC_50_ value than dill-H (470.62 ± 37.40 μg/mL). On the other hand, D-carvone reported the lowest IC_50_ value (275.7 ± 5.37 μg/mL) compared to apiole (388.35 ± 4.73 μg/mL), and DHC (>1 mg/mL). Previously, IC_50_ > 200 μg/mL was reported for the ethanolic dill extract [42]. The IC_50_ value for carvone obtained in our experiment was lower than previously obtained values of 835.2 μg/mL (5.56 mM) [43] but was similar to 277.9 μg/mL (1.85 mM) [44]. The conjugated double bonds present in carvone are responsible for better AChE inhibition [45]. It can be suggested that the anti-AChE activity of the extracts is the result of the synergistic action of several compounds present. Previously, essential oils from dill showed 100% inhibition, while carvone and DHC displayed only 23.6% and 31.1% AChE inhibition at 1 mg/mL [46]. Conversely, no AChE inhibition was observed in the aerial parts of the dill plant grown under organic and conventional agricultural conditions [47].

In silico studies predicted the binding energy of −5.6 and −5.9 kcal/mol for carvone and apiole, respectively [48]. In another study, carvone displayed a similar binding energy value (−7.7 kcal/mol) to galantamine, the positive inhibitor control for AChE [31]. Carvone was also reported as a competitive inhibitor of AChE (bovine erythrocyte) [44]. The molecular docking study revealed that carvone binds to the most important region of the AChE active site, interacting with esteratic site residues (Ser_203_, His_447_), and anionic site amino acids (Trp_86_, Tyr_133_, Tyr_337_, Phe_338_) responsible for catalytic action and substrate binding, respectively. It exhibited additional hydrogen bond interactions with Tyr_337_ to create an anionic subsite [49]. Conversely, the aromatic moiety of apiole interacted with Tyr_341_ and Trp_286_ at the active site of human AChE forming a π-bond. Additionally, the methylenedioxy group of apiole formed a hydrogen bond with the phenolic hydroxyl group of Tyr_124_ while the 2-methoxy group of apiole interacted with the peptide bond between Val_294_–Phe_295_ of the enzyme [50]. The above reports support our results for the competitive inhibition observed for dill, carvone, and apiole. In our study, V_max_ (the maximal reaction velocity when the enzyme is saturated with its substrate) remains unchanged (2.975 μmole/min/mg), but K_m_ (the concentration of the substrate that permits the enzyme to achieve half V_max_) increased in the presence of the inhibitor, indicating a competitive inhibition. An increased K_m_ value in the presence of an inhibitor reduces the binding affinity of the enzyme for the substrate requiring a higher concentration of the substrate to achieve V_max_. Since the competitive inhibitors can only bind to the free enzyme (E) and not to the enzyme–substrate (ES) complex, they cannot disturb ES catalysis, thus V_max_ is unaffected. K_i_ (inhibitor constant) determines the potential of the inhibitor and is described as the concentration required to produce half maximum inhibition. A smaller value of K_i_ indicates a stronger binding. In our study, the Ki value for carvone (270 μg/mL or 1.79 mM) was the lowest suggesting its strong binding to the enzyme. Previously, a lower K_i_ value (0.68 mM) was reported for carvone by Grundy et al. [51].

Formation and accumulation of amyloid plaques are serious pathological features of several diseases, including neurodegenerative diseases like AD and PD. Previously, aqueous [52], methanolic dill leaf extract [53], and dill tablet [26] markedly inhibited protein aggregation, which can generate amyloid cross-*β* structure and consequently affect protein structure and stability [53]. In an earlier report, hot water extract of dill inhibited amyloid-like fibril formation by approximately 10% [54]. Inhibitor binding can also alter the secondary structure of amyloid fibrils to less ordered or non-beta sheet structures without changing the morphology of amyloid aggregates or inhibiting fibril formation [55]. Additionally, compounds interacting with the central domain of Aβ inhibit oligomerization [56]. In our study, dill extracts and pure bioactives displayed a lower, but significant, Aβ fibrilization inhibition compared to the control. In the ThT assay, all pure compounds and dill-EA displayed similar activity while dill-H displayed lower inhibition. This difference might be due to the lower ratio of these bioactives (DHC: carvone: apiole:: 1: 1.3: 2.6) compared to dill-EA (DHC: carvone: apiole:: 1: 1.4: 5.4). In MDS assay, DHC was more effective in inhibiting oligomerization compared to others. DHC is the degradation product of carvone produced by hydrogenation. The better inhibition exhibited by DHC indicates the importance of hydrogenation in inhibition. The lower concentration of bioactives in the extracts might be responsible for the lower inhibition exerted by the extracts. The aromatic compounds interact with diphenylalanine’s π-stacking to exercise anti-amyloidogenic activity [57]. Consequently, it is reasoned that aromatic compounds in the extract stabilize the protein β structure through π-stacking or hydrophobic interaction [6]. According to the classification of compounds that inhibit Aβ assembly [58], our compounds are Class II inhibitors that stabilize Aβ conformation and do not promote oligomer of fibril formation.

The imbalance between the generation and detoxification of free radicals generates oxidative stress in the system, which is associated with the pathogenesis of various diseases including NDs. At low concentrations, ROS serves as a secondary messenger in cell signaling but at higher concentrations, it harms cellular components. Therefore, alleviating ROS provides a therapeutic approach to the treatment of diseases. In this regard, several plants have been identified that can reduce oxidative stress [59]. In the present study, dill extracts and bioactives displayed dose-dependent neuroprotection by reducing the ROS, protecting MMP, and restoring GSH/GSSG balance, most likely by antioxidant activity of the phytocompounds. The better neuroprotective effect exerted by dill extract might be due to the synergistic effect of major bioactive components. Additionally, the better neuroprotection displayed by dill-EA could be due to a higher content of apiole. In ROS assay, apiole, and DHC displayed significant ROS reduction at higher concentrations (10 and 30 μg/mL), while carvone also reduced ROS at lower (1 μg/mL) concentrations. Better ROS reduction by carvone could be due to its antioxidant [60,61,62] and anti-inflammatory actions involving the NF-kB signaling pathway [63]. In restoring MMP, the pure compounds performed better than the extracts, which might be due to the lower concentration of these bioactives in the extracts.

The slightly better significant activity displayed by dill-EA than dill-H in restoring glutathione levels might be due to a better profile of bioactives in it. The pretreatment of cells with dill restored levels of GSH/GSSG by promoting homeostatic redox control. A higher GSH: GSSG value than the control could be due to the de novo synthesis of GSH [64]. Moreover, sometimes flawed protection of free-SH groups of GSH during sample preparation might result in non-specific interactions, affecting the total concentration of free GSH [65]. Carvone displayed more significant results than apiole and DHC in reducing lipid peroxidation, which is again attributed to its antioxidant and anti-inflammatory nature [30,66].

Our results are supported by previous studies where ethanolic [67], aqueous [68], and hydroalcoholic [19] dill extracts improved cognition by inhibiting AChE, reducing MDA, and restoring the levels of protective enzymes in the antioxidant system [67,68]. In another study, a mixture of dill and *Ocimum* extract improved cognition by reducing stress and Aβ levels in the hippocampus [69] while methanolic extract provided neuroprotection in Aβ-induced PC12 cells [70].

An essential characteristic for a suitable neuroprotective agent to reach the target in CNS is its BBB permeability. Another pathway is the gut–microbiota–brain axis, by which orally administered phytocompounds may alter brain activity. While it is unclear if brain tissues have receptors or transporters for polyphenols or other phytochemicals, drugs targeting multiple sites are promising as a potential treatment for diseases with multifactorial etiology. Additionally, the possibility of secondary binding of phytocompounds to the targets cannot be denied [71]. Such extra-CNS actions have a slower effect than the direct interactions with the targets in CNS.

## 4. Materials and Methods

### 4.1. Chemicals

Acetylcholinesterase (*Electrophorus electricus*, Type VI-S), apiole, ascorbic acid, 2,2′-azinobis-(3-ethylbenzothiazoline-6-sulfonic acid) (ABTS), acetyl thiocholine chloride, 2,2-diphenyl-1-picrylhydrazyl (DPPH), 5,5′-Dithiobis(2-nitrobenzoic acid) (DTNB), Dihydrocarvone, 2′,7′-dichlorofluorescin diacetate (DCFDA), Folin–Ciocalteu reagent (FCR), galantamine, gallic acid, hydrogen peroxide, RIPA (Radio-Immunoprecipitation Assay) Buffer, Thioflavin T (ThT), 2,4,6-tripyridyl-s-triazine (TPTZ), tetramethylrhodamine, ethyl ester (TMRE) were bought from Sigma-Aldrich (St. Louis, MO, USA). Carvone was purchased from Santa Cruz Biotechnology (Dallas, TX, USA), and Aβ_1–42_ (Aggresure™) was bought from AnaSpec (Fremont, CA, USA). The WST-8 kit was purchased from Roche Diagnostics GmbH (Mannheim, Germany). Aβ_1–42_ for MDS (GenicBio Inc., Shanghai, China), purified anti-Aβ_1–16_ antibody (Biolegend, San Diego, CA, USA), and the horseradish peroxidase (HRP)-conjugated W_0–2_ monoclonal antibody (Peoplebio Inc., Seongnam, Republic of Korea). Thermo Fisher Scientific (Waltham, MA, USA) was the source for 3,3′,5,5′-Tetramethylbenzidine solution (TMB), fetal bovine serum (FBS), kanamycin, penicillin, and phosphate-buffered saline (PBST). Dulbecco’s modified Eagle’s medium (DMEM) was supplied by Gibco (Thermo Fisher, Seoul, Republic of Korea). All organic solvents of HPLC grade were purchased from Sigma-Aldrich (St. Louis, MO, USA).

### 4.2. Plant Material and Extraction

The pre-weighted (25 g) dried seeds of dill (Expat Mart, Seoul, South Korea) were ground using a pestle mortar and sequentially extracted by increasing polarity in the following order: (i) hexane and (ii) ethyl acetate. To a conical flask containing the sample, hexane (polarity = 0.009) was added and subjected to mild shaking for 8 h. They were first filtered with muslin cloth and then through Whatman No. 1 filter paper. The residue was further extracted twice using the same fresh solvent and all the filtrates were pooled together. The resulting residue was air-dried and further extracted with ethyl acetate (polarity = 0.228) using the same procedure. Finally, the solvent was removed using a rotatory evaporator (EYELA, Japan) under reduced pressure and low temperature. The fractions were weighed and kept at 4 °C until additional experiments.

### 4.3. Gas Chromatography-Mass Spectrometry (GC-MS) Method

The sample was separated on a fused silica capillary column (DB-5ms UI, 30 m × 0.25 mm i.d., film thickness 0.25 μm, Agilent, Santa Clara, CA, USA) installed on GCMS-QP2020 (Shimadzu, Kyoto, Japan). The oven temperature was programmed as isothermic at 60 °C for 2 min, 100 °C at 4 °C/min, 290 °C at 10 °C/min, and finally isothermic for 10 min. The split injection mode (1:10) was used. The carrier gas was helium at a constant flow rate (1 mL/min). The injection port, ion source, and interface temperatures were 280, 280, and 150 °C, respectively. The ionization energy was 70 eV. The mass spectra were obtained in full scan mode (40–700 AMU). The sample (1 μL, 1 mg/mL) was auto-injected into the GC-MS. The unknown compounds were identified by matching known compounds in the National Institute of Standards and Technology (NIST) library.

### 4.4. Determination of Total Phenolic and Flavonoid Content

The total phenol content (TPC) of extracts (1 mg/mL) was determined colorimetrically using the Folin–Ciocalteu reagent with slight modification [23] in the original method [72]. Gallic acid standard (10–100 μg/L) was used for calibration. Total phenolic content was expressed as mg gallic acid equivalents (GAE) per g of plant extract.

The total flavonoid content of extracts (1 mg/mL) was determined by aluminum chloride colorimetric assay with slight modification [23] in a previously reported method [72]. Quercetine standard (10–100 μg/mL) was used for calibration. Total flavonoid contents were expressed as mg quercetin equivalents (QE) per g of plant extract.

### 4.5. Determination of Antioxidant Capacity

(i) 2,2′-Azino-Bis (3-Ethylbenzothiazoline-6-Sulfonic Acid) [ABTS] Radical Scavenging Assay

The free radical scavenging activity of the extracts and phytocompounds at 1 mg/mL was measured spectrophotometrically in a plate reader (Synergy-H1 BioTek, Agilent, Santa Clara, CA, USA) by modifying a previous method [73] to suit the 96-well plate format [23]. Quercetin served as a positive control, methanol as a negative control, and extract without ABTS as blank. A standard curve for quercetin (1–30 μg/mL) was prepared for calibration and the results were expressed in milligram equivalents of quercetin per milligram of dry weight. The percentage of inhibition of ABTS^+*•*^ was calculated as:

% RSA = (Ab *−* Ae/Ab) *×* 100

where Ab = absorbance of the blank and Ae = absorbance of the extract.

(ii) Free Radical Scavenging by 2,2-Diphenyl-1-Picrylhydrazylhydrate (DPPH) Radical Assay

DPPH radical scavenging capacity of the extracts and phytocompounds at 1 mg/mL was measured with slight modification [74]. The absorbance was monitored at 515 nm (Multimode reader, Synergy-H1 BioTek, Agilent, Santa Clara, CA, USA) using ascorbic acid (0.1–10 μg/mL) as a standard. Radical scavenging activity (RSA) was calculated as:

% RSA = (Ab *−* Ae/Ab) *×* 100

where Ab = absorbance of the blank and Ae = absorbance of the extract.

(iii) Ferric Reducing Antioxidant Potential (FRAP) Assay

FRAP assay was used to assess the metal-chelating ability of the extracts and phytocompounds with slight modification [23] in a previously reported method [75]. For the assay, the extract (1 mg/mL) was incubated with 200 μL of FRAP reagent, and the reduction of ferric tripyridyltriazine was monitored at 593 nm (Multimode reader, Synergy-H1 BioTek, Agilent, USA). Ascorbic acid (2 μg/mL) was used as a positive control. FRAP values of the extracts and phytocompounds were calculated from the standard curve of FeSO_4_ (15–250 μM) and expressed as μM Fe^2+^/g.

### 4.6. Acetylcholinesterase Inhibitory Activity

The AChE activity was examined by slight modifications [23] in Ellman’s method [76]. The extracts and phytocompounds were incubated for 15 min with AChE and 10 mM ATCC at 37 °C. The reaction was stopped by DTNB (15 mM), and the absorbance was measured at 412 nm (Multi-mode plate reader, Synergy-H1 BioTek, Agilent, USA). Galantamine was used as the positive control. The percent inhibition was calculated as:

Percent Inhibition (I%) = [(Ao *−* Ac) *−* (Bi *−* Bc)]/(Ao *−* Ac) *×* 100

where Ao is the absorbance without inhibitor; Ac is the negative control without inhibitor; Bi is the absorbance with inhibitor; and Bc is the negative control with inhibitor. The IC_50_ values were calculated using GraphPad Prism 10. Lineweaver–Burk plot was used to prepare the inhibition curves (with and without extract/phytocompound) using GraphPad Prism 10. The kinetic parameters were calculated from a non-linear fit (Michaelis–Menten equation) in GraphPad Prism 10.

### 4.7. Anti-Aβ_1–42_ Oligomerization and Fibrilization Activity

The anti-Aβ_1–42_ oligomerization and fibrilization activity of the extracts/bioactives were measured by Multiple Detection System (MDS) and ThT assay, respectively [6]. Briefly, the extracts and phytocompounds were incubated with Aβ_1–42_ at RT for different time points (0 h, 2 h, and 4 h). The samples were incubated on an anti-β-amyloid pre-coated plate for 1 h at RT. An HRP-conjugated W_0–2_ monoclonal antibody was added, and the plate was kept at RT for 30 min. Later, TMB was added, and the plate was incubated for 15 min at RT. The absorbance was read at 450 nm using a microplate reader (Victor3, PerkinElmer, Shelton, CT, USA)

The anti-Aβ_1–42_ fibrilization activity of the extracts/bioactives was monitored using a ThT assay [6]. The samples were incubated in the presence/absence of Aβ_1–42_ at 37 °C for 24 h. The samples were incubated with 100 μM ThT at 37 °C for 15 min. The fluorescence was monitored at Ex 450 nm/Ems 490 nm (Synergy-H1 BioTek, Agilent, Santa Clara, CA, USA). For the control, phenol red (50 μM) was used. The Aβ_1–42_ aggregation inhibition was calculated as follows:

Percent Inhibition (%) = [(1 − Fi/Fc) × 100]

where Fi and Fc are the fluorescence intensity with and without the inhibitors, respectively.

### 4.8. Cell Culture

Human neuroblastoma SH-SY5Y cells (ATCC CRL-2266, Manassas, VA, USA) were maintained in Dulbecco’s modified Eagle’s medium (DMEM, Gibco) supplemented with 10% fetal bovine serum (FBS), 1% kanamycin, and 1% penicillin (Thermo Fisher Scientific, Waltham, MA, USA) at 37 °C with 5% CO_2_, and a 95% humidified atmosphere in the incubator. The experiments were executed at 80–90% cell confluency.

#### 4.8.1. Cell Viability Assay

The cells were seeded (1 *×* 10^4^ cells/well) in 96-well sterile plates and pre-treated with various extract concentrations (1, 10, 30 µg/mL) for 24 h. The extracts and phytocompounds were removed and incubated for 2 h with 10% WST-8 reagent (Roche, Grenzach-Wyhlen, Germany) as described previously [23]. The absorbance was determined at 450 nm in a multi-plate reader (Synergy-H1 BioTek, Agilent, USA). The percent cytotoxicity was calculated as:

Cytotoxicity % = (Ac − At)/(Ac) *×* 100

where Ac = absorbance of the control cells, At = absorbance of the treated cells.

The plot of percent cytotoxicity versus sample concentration was used to calculate the extract concentration that killed 50% of the cells (IC_50_).

#### 4.8.2. Neuroprotection Assay

The neuroprotective effect of extracts and phytocompounds on H_2_O_2_-induced oxidative stress in SH-SY5Y by a previously described method [23]. The cells (1 *×* 10^4^ cells/well) were seeded in a 96-well sterile plate. After stabilization, the cells were pre-treated with the extracts and phytocompounds for 24 h. The extracts and phytocompounds were removed and treated with H_2_O_2_ (100 μM) for 6 h. A solvent control, H_2_O_2_ alone, and extract alone treatments were also included. After incubation, the % cell viability was measured using WST-8 reagent in triplicate experiments.

#### 4.8.3. Measurement of Intracellular Reactive Oxygen Species (ROS)

The cells (1 × 10^4^ cells/well) were seeded in a 96-well sterile plate, after which they were pre-treated with the extract for 12 h. The extracts and phytocompounds were removed, followed by a 4 h treatment with H_2_O_2_ (100 μM) and H2DCFDA (25 μM) for another 2 h in the dark at 37 °C [23]. The fluorescence intensity (Ex 495 nm, Ems 520 nm) was measured by a microplate reader (Synergy-H1 BioTek, Agilent, USA). The ROS was calculated as a percentage of the untreated control cells (100%) in triplicate measurements.

#### 4.8.4. Mitochondrial Membrane Potential (ΔΨm) Assay

The mitochondrial membrane potential was measured using the tetramethylrhodamine, methyl ester (TMRE) staining method [23]. The cells (1 *×* 10^4^ cells/well) were seeded in a 96-well sterile plate; after which, they were pre-treated with the extract for 12 h. The extracts and phytocompounds were removed, followed by a 2 h treatment with H_2_O_2_ (200 μM). A 1 μM amount of TMRE was added to the cells and incubated for 1 h at 37 °C. The fluorescence was assessed (Ex 549 nm, Ems 575 nm) using a microplate reader (Synergy-H1 BioTek, Agilent, USA). The ΔΨm was calculated as a percentage of the untreated control cells (100%) in triplicate measurements.

#### 4.8.5. Antioxidant Parameters in Cell Lysate

The cells (5 × 10^4^ cells/well) were seeded in a 6-well sterile plate and incubated for 18–24 h. After stabilization, cells were pre-treated with the extracts and phytocompounds for 24 h before 6 h incubation with H_2_O_2_ (100 μM). The culture media was removed, and the cells were washed with cold PBS (1X). The cells were placed on ice and incubated with pre-chilled RIPA buffer for 10 min. Transfer the lysate in microfuge tubes and centrifuge (Labogene 1730R, BMS, Paju-si, Republic of Korea) for 10 min at 20,000× *g*, 4 °C. The supernatant was collected and stored at −80 °C.

##### Protein Estimation

The protein concentration in the samples was measured using the BCA protein estimation kit (Thermo Scientific, Waltham, MA, USA). The BSA standard (10–1000 μg/mL) was used to calculate protein concentration in the unknown samples.

##### Estimation of Glutathione

The concentrations of GSH (reduced) and GSSG (oxidized) in the lysate were measured fluorometrically using a previously described [23] and the fluorescence was recorded at 350/420 (Ex/Ems) in a microplate reader (Synergy-H1 BioTek, Agilent, Santa Clara, CA, USA).

##### Estimation of Malondialdehyde (MDA)

MDA levels were measured using TBA: TCA reagent as described previously [23]. In the reaction, MDA reacts with two molecules of thiobarbituric acid (TBA) to give a pink pigment that absorbs at 532 nm. The standard curve of MDA (1–100 μM) was used to calculate lipid peroxidation in the lysate.

### 4.9. Data and Statistical Analysis

Statistical analysis was established by a one-way ANOVA followed by Dunnett’s post hoc test. Data are registered as the mean *±* SEM of three sets of experiments. The symbols ^####^, **** represents *p* < 0.0001, ^###^, *** represents *p* < 0.001, ^##^, ** represents *p* < 0.01, and ^#^, * represents *p* < 0.05. The symbol ^#^ indicates significance compared to the H_2_O_2_ control while * indicates significance compared to the untreated control. The IC_50_ values were determined using non-linear regression. The V_max_ and K_m_ were calculated from a Michaelis–Menten plot drawn using a non-linear plot (GraphPad Prism 10). Lineweaver–Burk plots were drawn using linear regression analysis (GraphPad Prism 10). The K_i_ values were calculated from the formula IC_50_ = K_i_ (1 + [S]/K_m_).

## 5. Conclusions

The study provides a phytochemical basis for some of the effects of dill extracts and their main phytocompounds on neuroprotection. In summary, we evaluated the neuroprotective potential of dill extracts and main bioactive compounds in H_2_O_2_-induced oxidative stress in human neuroblastoma SH-SY5Y cell lines and biochemical studies. The dill extract and phytocompounds significantly provided neuroprotection by reducing oxidative stress, restoring MMP, and re-establishing redox homeostasis in the cells, indicating their antioxidant potential at varying concentrations (0.1–30 μg/mL). The antioxidant potential of dill is the most reasonable explanation for the neuroprotective effect in the cells. In addition, they exerted moderate anti-AChE activity and competitively inhibited the enzyme. The anti-AChE activity of pure compounds was relatively lower than the extracts, except for DHC. They also showed mild anti-Aβ oligomerization and good anti-Aβ fibrilization activity, stabilizing Aβ conformation to prevent oligomer/fibril development. The structural modifications in these lead compounds will be helpful in further refinement of activities. This attractive alternative multitargeted neuroprotective approach would be beneficial in the development of cost-effective drugs for multifactorial ailments like NDs, especially AD. However, despite promising results, additional research is needed to explore their role in neuroprotection especially in human subjects.

## Figures and Tables

**Table 1:** Kinetic parameters for AChE inhibition by dill and its phytocompounds.

	Vmax (μmole/min/mg)	Km (mM)	Ki (µg/mL)	Type of Inhibition
No inhibitor	2.975	23.43		
Dill-H (100 μg/mL)	3.472	31.94	463.36	Competitive
Dill-H (200 μg/mL)	3.710	39.14	464.68	
Dill-EA (100 μg/mL)	2.921	27.39	495.05	Competitive
Dill-EA (200 μg/mL)	3.512	38.21	497.58	
Apiole (100 μg/mL)	3.551	31.02	382.18	Competitive
Apiole (200 μg/mL)	3.712	34.59	382.81	
Carvone (100 μg/mL)	2.900	25.12	270.31	Competitive
Carvone (200 μg/mL)	3.141	25.28	270.34	

## Data Availability

Data are contained within the article. Additional information is provided as Supplementary Materials.

## Supplementary Materials

The following supporting information can be downloaded at: https://www.mdpi.com/article/10.3390/ijms25137104/s1.
