# Supplementary material for: Neuroprotection by Anethum graveolens (Dill) Seeds and Its Phytocompounds in SH-SY5Y Neuroblastoma Cell Lines and Acellular Assays
Source: Int J Mol Sci. 2024 Jun 28;25(13):7104. doi: 10.3390/ijms25137104 (PMC11241255; doi:10.3390/ijms25137104)
Supplement: Supplementary file 1 [file ijms-25-07104-s001.zip › ijms-3035723-supplementary.pdf]

Supplementary: Neuroprotection by *Anethum graveolens (Dill)* Seeds and its Phytocompounds in SH-SY5Y Neuroblastoma Cell Lines and Acellular Assays

Himadri Sharma, Hyewon Yang, Niti Sharma* and Seong Soo A. An*

Department of Bionano Technology, Gachon Bionano Research Institute, Gachon University,

1342 Seongnam-daero, Sujung-gu, Seongnam-si 461-701, Gyeonggi-do, Republic of Korea.

*Correspondence: **nitisharma@gachon.ac.kr (N.S.); seongaan@gachon.ac.kr (S.S.A.A.);**

Tel.: +82-31-750-8591 (N.S.); +82-31-750-8755 (S.S.A.A.)

Figure S1: GC-MS profiling and the phytoconstituents identified in (A) dill-H and (B) dill-EA extracts.

A.

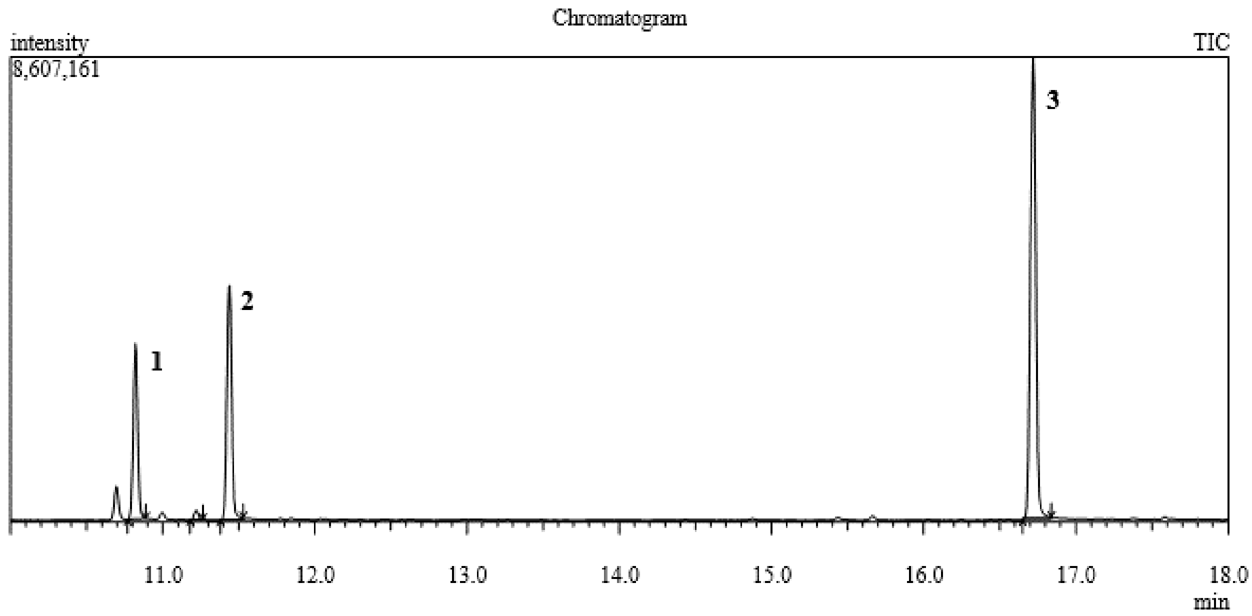

Major Peak No.	R Time	Area	Area (%)	Height	Height (%)	Name
**1**	**10.821**	**6601292**	**17.29**	**3243576**	**19.90**	**Cyclohexanone, 2-methyl-5-(1-methylethenyl)-**
	11.221	348267	0.91	176863	1.08	Neodihydrocarveol
**2**	**11.438**	**9518814**	**24.94**	**4328511**	**26.55**	**D-Carvone**
**3**	**16.719**	**21704057**	**56.86**	**8552410**	**52.46**	**Apiol**
		38172430	100.00	16301360	100.00	

B

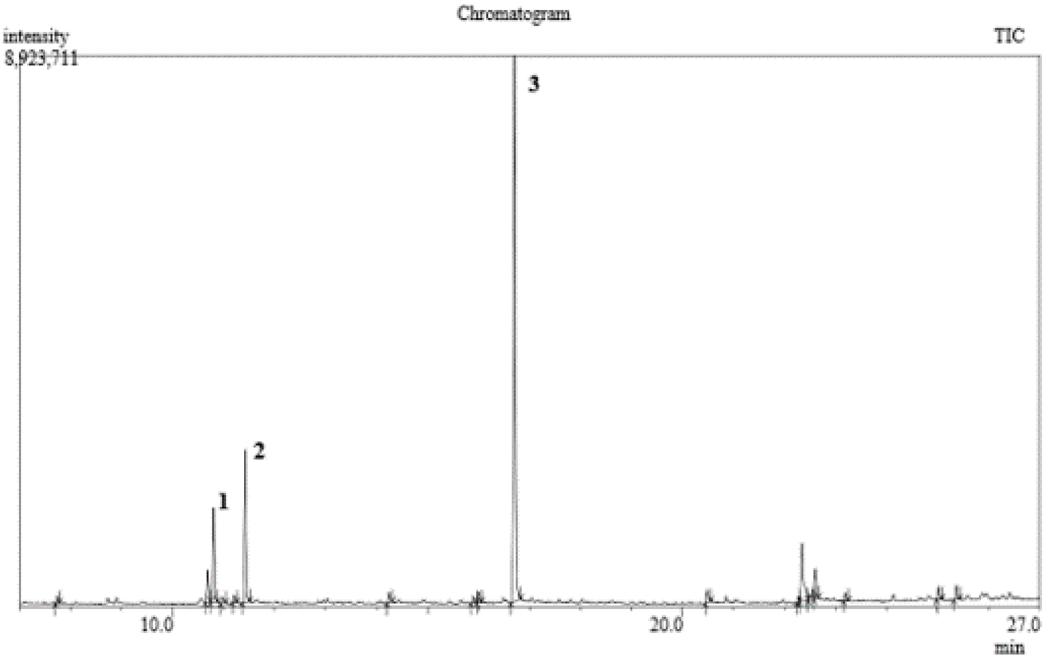

Major peak no.	R. Time	Area	Area (%)	Height	Height (%)	Name
	7.744	203259	0.50	114477	0.69	D-Limonene
	10.687	1037084	2.54	517157	3.14	Cyclohexanone, 2-methyl-5-(1-methylethenyl)-
**1**	**10.812**	**3105456**	**7.60**	**1525273**	**9.26**	**Cyclohexanone, 2-methyl-5-(1-methylethenyl)-**
	10.989	200979	0.49	95756	0.58	Cyclohexanol, 2-methyl-5-(1-methylethenyl)
	11.212	226067	0.55	114015	0.69	Cyclohexanol, 2-methyl-5-(1-methylethenyl)
**2**	**11.427**	**5224943**	**12.78**	**2455453**	**14.90**	**D-Carvone**
	14.251	353626	0.87	163972	1.00	Carvone oxide, cis-
	15.890	244018	0.60	100492	0.61	3',5'-Dimethoxyacetophenone
	16.006	382504	0.94	166240	1.01	(E)-Tetradec-2-enal
**3**	**16.710**	**22861945**	**55.93**	**8831602**	**53.61**	**Apiol**
	20.479	478362	1.17	202659	1.23	l-(+)-Ascorbic acid 2,6-dihexadecanoate
	22.260	220862	0.54	78706	0.48	Pentadecanoic acid
	22.338	3446176	8.43	931768	5.66	Octadec-9-enoic acid
	22.545	621166	1.52	143534	0.87	trans,trans-9,12-Octadecadienoic acid, propy
	22.606	1023226	2.50	475582	2.89	(E)-9-Octadecenoic acid ethyl ester
	23.191	261275	0.64	127581	0.77	Eicosanal-
	25.025	437798	1.07	208481	1.27	Henicosanal
	25.366	546607	1.34	221854	1.35	1,2-Propanediol, 3-benzyloxy-1,2-diacetyl-
		40875353	100.00	16474602	100.00	

Figure S2. Lineweaver-Burk plots of AChE in the presence of 100 μg/mL and 200 μg/mL of dill extracts (A-B), Apiole (C), and Carvone (D). The graphs were plotted using GraphPad Prism 10. Abbreviations: V: Velocity of enzyme-catalyzed reaction; S: Substrate

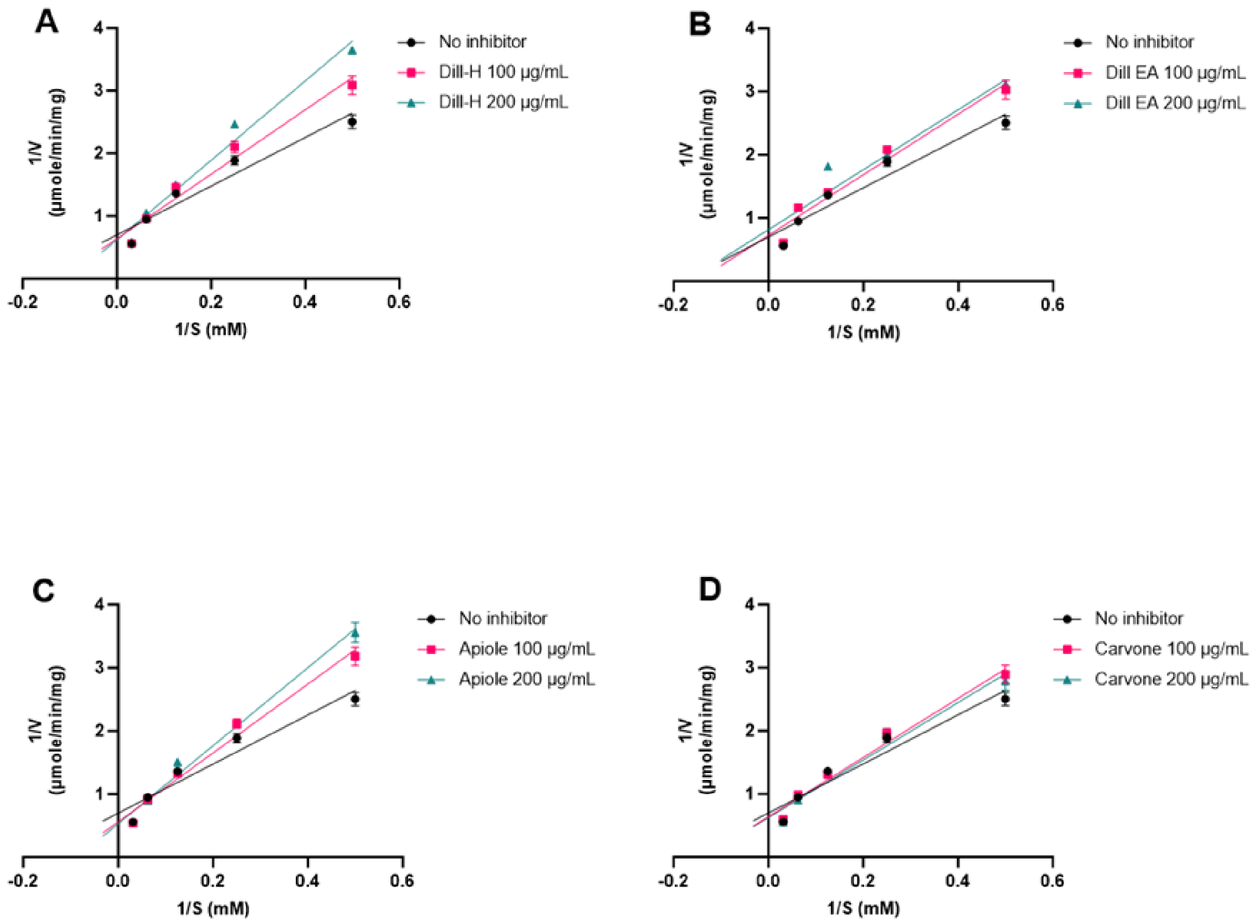

Figure S3. Cytotoxicity assay of dill extracts, apiole, carvone, and DHC on the SH-SY5Y cells. The cells were treated for 24 h with varying extract concentrations (1, 10, and 30 µg/mL). The cell viability was reported as the percentage of the control group (100%). All data are presented as mean ± SEM (n = 3). No significant difference was observed using one-way ANOVA followed by Dunnett's post hoc in the % of cell viability in the treated vs. the control group (no treatment).

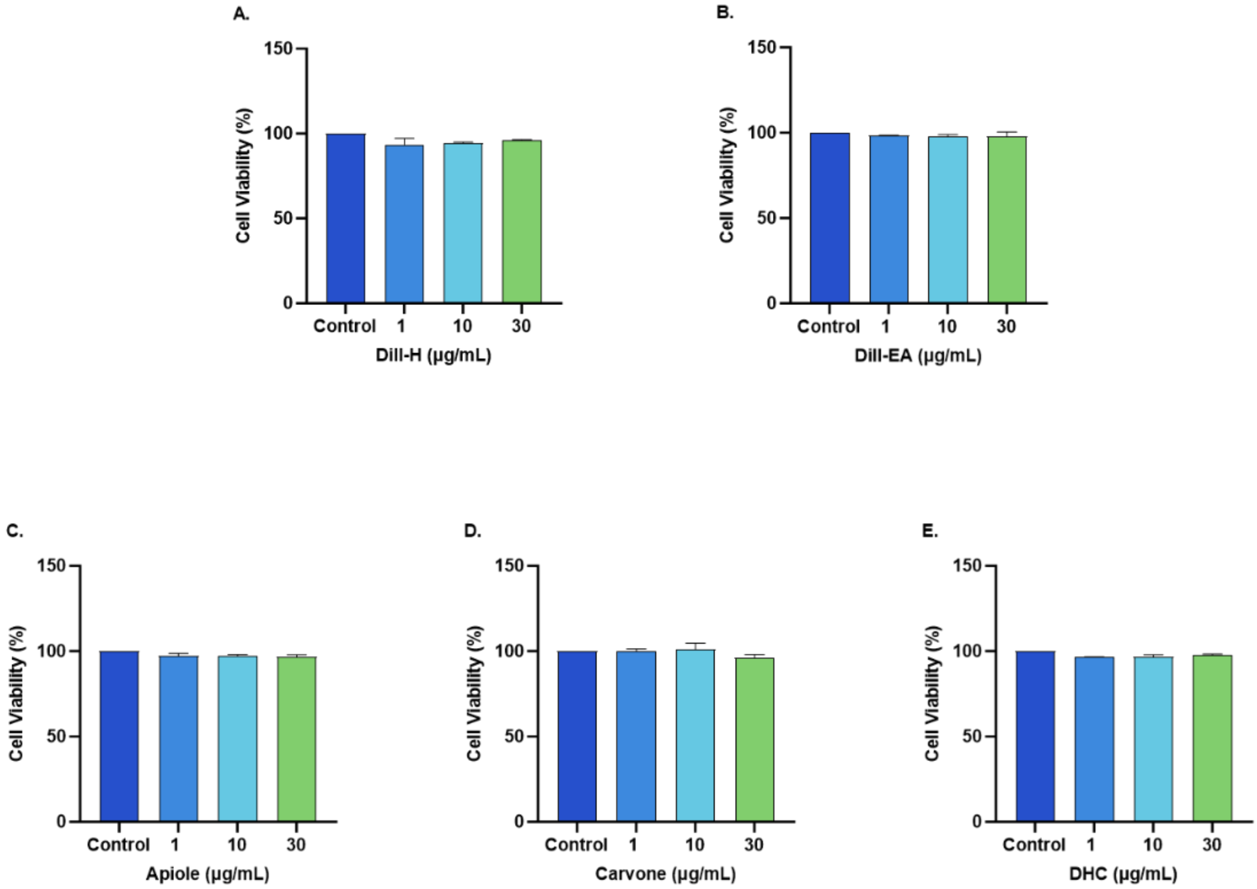

Figure S4: Neuroprotective effect of dill and its major photo compounds at 1 μg/mL and 30 μg/mL in the presence of 100 μM H_2O_2. The images were taken on JuLI Stage (JS160504-002) at 10X.

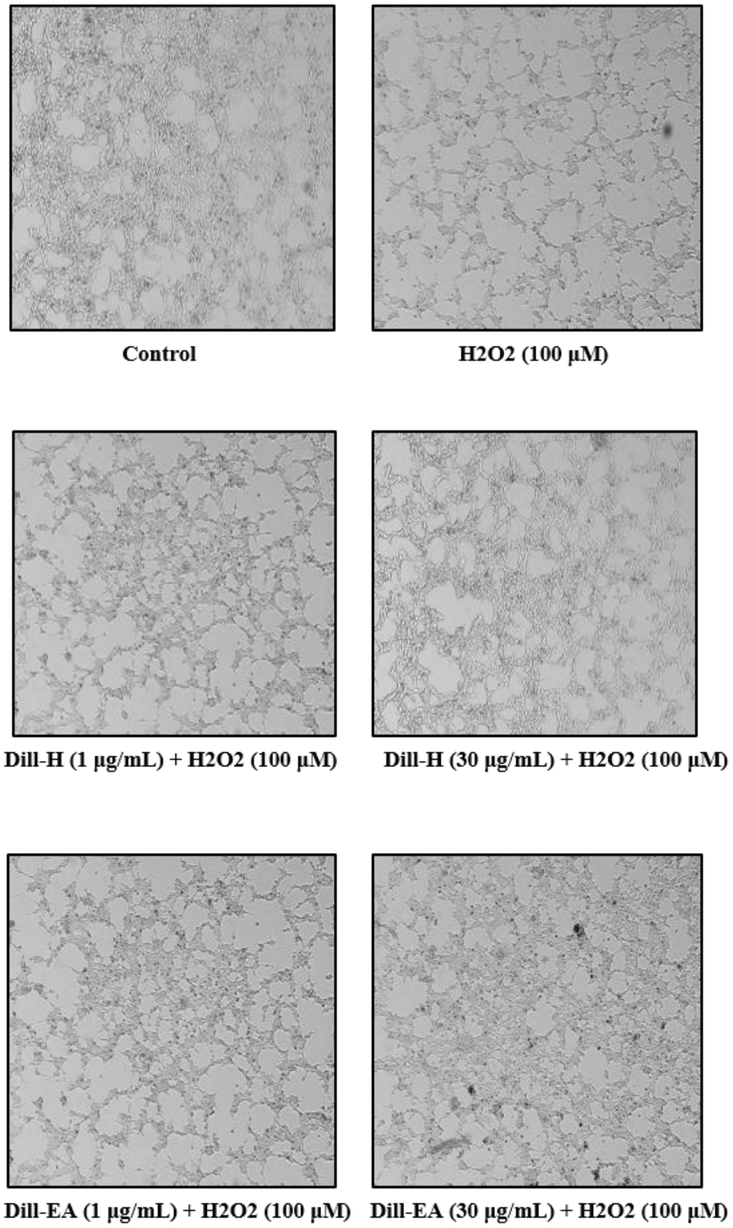

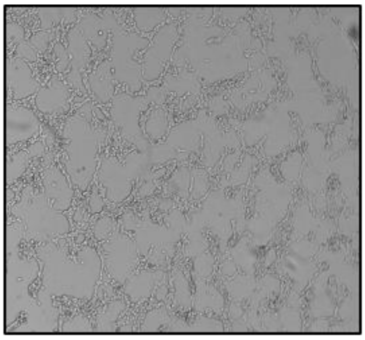

Apiole (1 μg/mL) + H2O2 (100 μM)

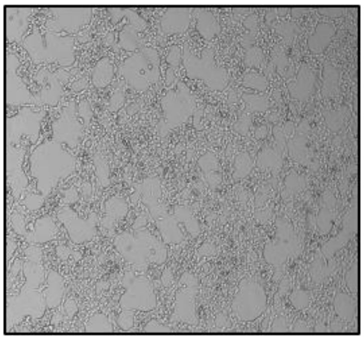

Apiole (30 μg/mL) + H2O2 (100 μM)

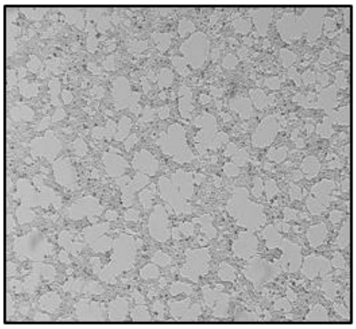

Carvone (1 μg/mL) + H2O2 (100 μM)

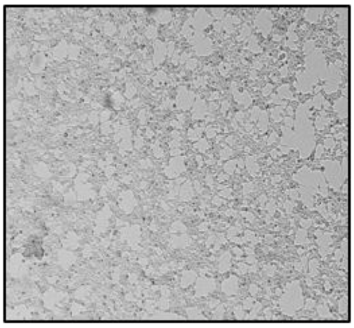

Carvone (30 μg/mL) + H2O2 (100 μM)

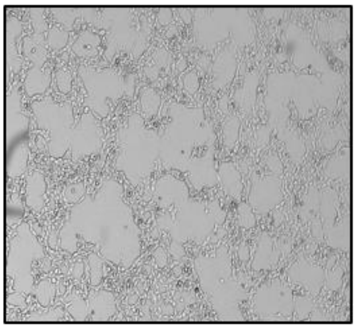

DHC (1 μg/mL) + H2O2 (100 μM)

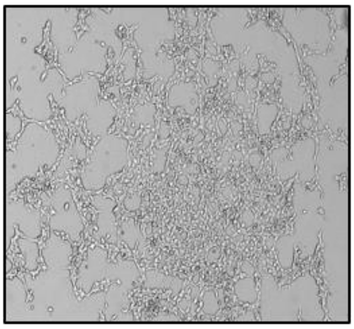

DHC (30 μg/mL) + H2O2 (100 μM)

Figure S5. Reduced glutathione (GSH) content in SH-SY5Y cell lysate exposed to 100 µM H_2O_2 for 6 h after 24 h of pre-treatment with dill extracts, apiole, carvone, and DHC. The results indicate GSH (µM/µg protein) levels in treated and control cells (untreated cells). Values are mean ± SEM (n = 3). The data were analyzed by one-way ANOVA followed by Dunnett's test. A significant difference */# ($p < 0.05$), **/## ($p < 0.01$), and ***/### ($p < 0.001$) was observed in comparison to untreated cells (*) and H_2O_2 treated cells (#).

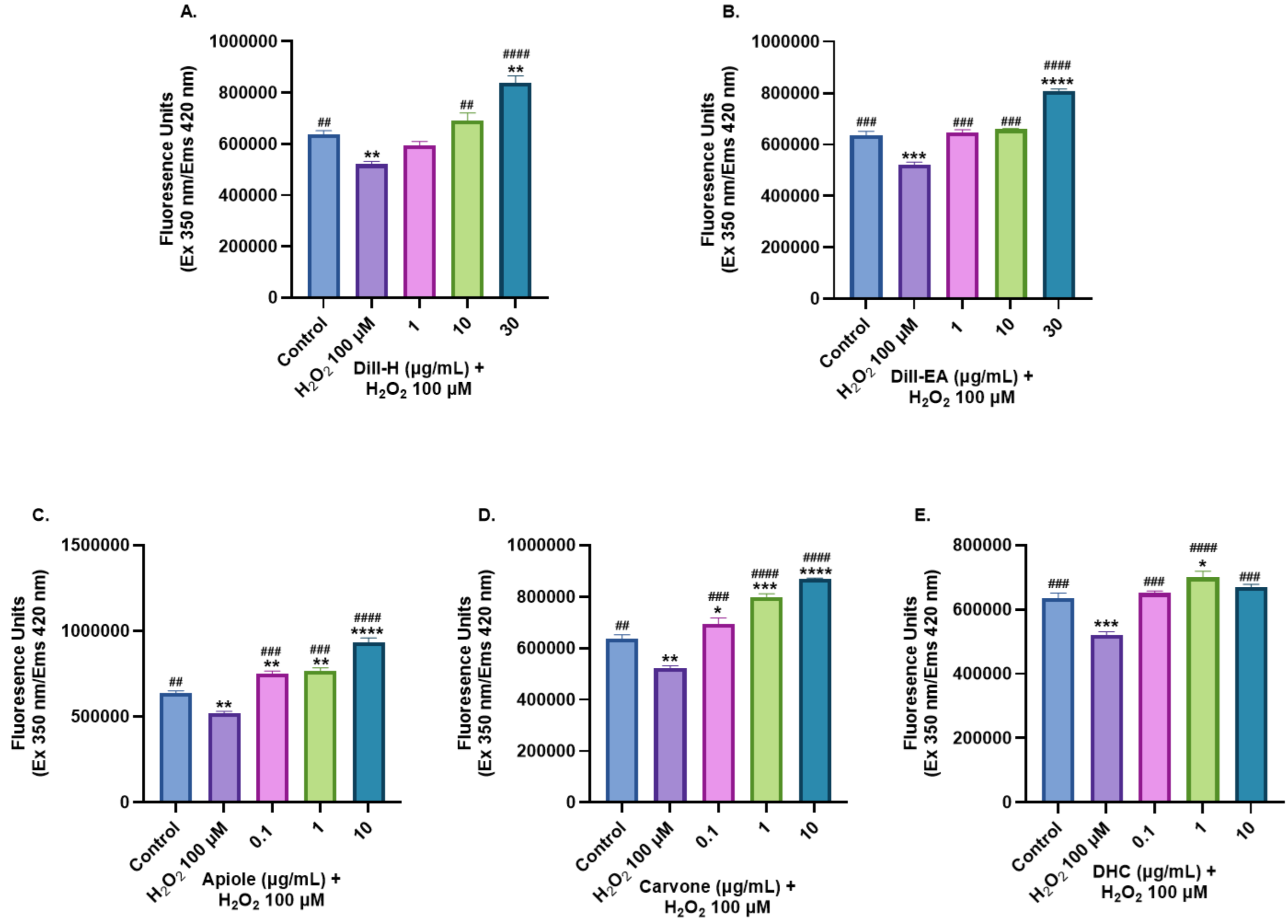

Figure S6. Oxidized glutathione (GSSG) content in the SH-SY5Y cell lysate exposed to 100 µM H_2O_2 for 6 h after 24 h of pre-treatment with dill extracts, apiole, carvone, and DHC. The results indicate GSSG (µM/µg protein) levels in treated and control cells (untreated cells). Values are mean ± SEM (*n* = 3). The data were analyzed by one-way ANOVA followed by Dunnett's test. A significant difference of */# ($p < 0.05$), and **/## ($p < 0.01$) was observed in comparison to untreated cells (*) and H_2O_2-treated cells (#).

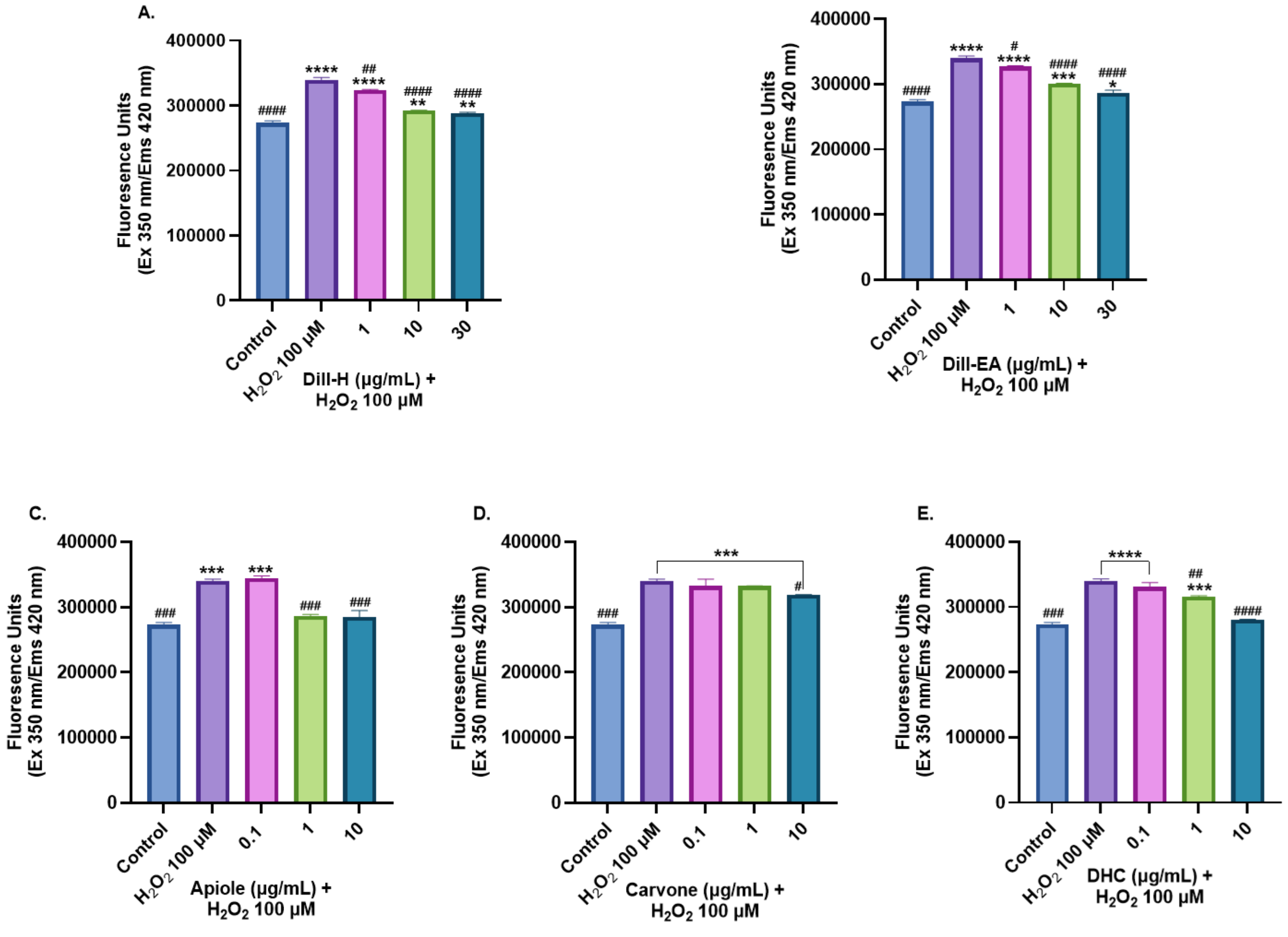

Table S1

Summary of Total Phenolic Content (TPC), Total Flavonoid Content (TFC), and Antioxidant Activity by 2,2′-Azino-bis (3-Ethylbenzothiazoline-6-Sulfonic Acid) [ABTS] Radical Scavenging Assay; 2,2-di-phenyl-1-picrylhydrazyl (DPPH) radical scavenging capacity assay; ferric reducing (FRAP) assay methods for dill-H and dill-EA extracts and phytocompounds. Values are expressed as mean ± SEM (n=3).

	TPC (mg GAE/g)	TFC (mg QE/g)	ABTS (% RSA)	DPPH (% RSA)	FRAP (µM Fe^{2+}/g)
**Dill-H**	16.20 ± 2.88	8.69 ± 2.05	37.17 ± 0.14	37.11 ± 0.23	68.48 ± 1.20
**Dill-EA**	43.55 ± 3.42	18.94 ± 3.45	45.72 ± 0.64	53.00 ± 0.71	103.00 ± 1.03
**Apiole**	-	-	20.71 ± 0.26	29.73 ± 2.27	42.41 ± 3.54
**Carvone**	-	-	20.02 ± 0.18	37.05 ± 0.25	43.25 ± 2.50
**DHC**	-	-	17.53 ± 0.47	36.85 ± 1.51	45.22 ± 0.72
